# Exploiting Curcumin Synergy With Natural Products Using Quantitative Analysis of Dose–Effect Relationships in an Experimental *In Vitro* Model of Osteoarthritis

**DOI:** 10.3389/fphar.2019.01347

**Published:** 2019-11-14

**Authors:** Angela D’Ascola, Natasha Irrera, Roberta Ettari, Alessandra Bitto, Giovanni Pallio, Federica Mannino, Marco Atteritano, Giuseppe M. Campo, Letteria Minutoli, Vincenzo Arcoraci, Violetta Squadrito, Giacomo Picciolo, Francesco Squadrito, Domenica Altavilla

**Affiliations:** ^1^Department of Clinical and Experimental Medicine, University of Messina, Messina, Italy; ^2^Department of Chemical, Biological, Pharmaceutical and Environmental Sciences, University of Messina, Messina, Italy; ^3^Department of Biomedical and Dental Sciences and Morphofunctional Imaging, University of Messina, Messina, Italy

**Keywords:** synergy, curcumin, flavocoxid, beta-caryophyllene, inflammation

## Abstract

**Introduction:** Drug combination is widely used to treat chronic inflammatory diseases. A similar strategy might be worth of interest to design plant-derived natural products to treat inflammatory conditions. Curcumin is a natural phenolic compound which shares anti-inflammatory activity with both flavocoxid, a flavonoid mixture of baicalin and catechin, and β-caryophyllene, a bicyclic sesquiterpene. The aim of this study was to investigate the synergy potential of curcumin with both flavocoxid and β-caryophyllene in human articular chondrocytes triggered with lipopolysaccharide (LPS), in an experimental *in vitro* model of osteoarthritis.

**Materials and Methods:** Human articular chondrocytes were stimulated with LPS alone or in combination with different treatments. Total RNA was extracted 4 h after treatment to study interleukin 1β (IL-1β), NF-κB, and STAT3 mRNA expression. A drug combination study was designed choosing 5 doses to demonstrate a synergistic effect of compounds, according to Chou and Talalay method. A median-effect equation was applied and finally, the combination index (CI) was used to clarify the nature of the compounds interaction (synergistic versus additive versus antagonistic inhibitory effects); CI < 1, CI = 1, and CI > 1 indicated synergistic, additive, and antagonistic effects, respectively.

**Results:** LPS prompted IL-1β expression. Curcumin, flavocoxid and β-caryophyllene suppressed IL-1β expression with different IC_50_. A synergistic action for the reduction of the inflammatory phenotype in human chondrocytes was observed for the combination curcumin-flavocoxid with a percentage from 10% to 90%, and for the combination curcumin-β-caryophyllene from 50% to 90%. IC_50_ doses of either flavocoxid, β-caryophyllene and curcumin alone or in combination were safe and did not affect cell vitality. Moreover, the same IC_50_ doses reduced the transcription factors NF-κB and STAT3 mRNA expression and interestingly the effects of the combinations were greater than the natural products alone, thus suggesting that the site where the synergy takes place could be at the signal transduction level.

**Discussion:** The results suggest that curcumin synergizes with either flavocoxid or β-caryophyllene, exerting an anti-inflammatory activity and thus strongly suggesting the potential of a dual combination of these compounds for the management of osteoarthritis and unmasking a new feature of these natural products.

## Introduction

The last decades have testified increased interest in active plants derived substances that have gained authorization and reached the market as approved medicines (Fürst and Zündorf, 2015). Interestingly, the use of natural medicine has grown and a huge amount of people around the globe seeks remedy for their health problem in phytotherapy (Thomford et al., 2018a). This scenario has prompted the interest of the pharmaceutical industry in the research and development of new medicines to treat chronic diseases. Nowadays, about 28% of all modern drugs are produced from natural sources, thus pointing out the enormous medicinal potential of plant derived compounds (Fürst and Zündorf, 2015; Thomford et al., 2018a). Precision medicine is an emerging approach for diseases treatment and in agreement with this, the proposal of an individualized therapy with natural products has also attracted the interest of scientists in this field (Thomford et al., 2018b).

Natural products have been used for the management and treatment of osteoarthritis (OA) (Henrotin and Mobasheri, 2018). Curcumin is a naturally occurring phenolic compound extracted as a yellow pigment from spice turmeric (*Curcuma longa L.*). It inhibits the inflammatory response by suppressing NF-κB signaling in rat chondrocytes and modulates collagen deposition, matrix metalloproteinase-13, and cell proliferation throughout the reduction of interleukin-1β (IL-1β) (Wang et al., 2017). Furthermore, it has been shown that the phenolic natural compound prevents articular chondrocytes from apoptosis triggered by sodium nitroprusside (Zhao et al., 2018). The systemic therapeutic potential of curcumin has been also demonstrated in OA using an OA rat model (Ratanavaraporn et al., 2017), while the clinical efficacy of this compound has been confirmed in patients suffering from the inflammatory pathology (Liu et al., 2018).

Beta-caryophyllene (β-caryophyllene [BCP]) is a plant-derived FDA approved natural product with interesting therapeutic potential. It is a bicyclic sesquiterpene found in copaiba (*Copaifera* spp) and marijuana/hemp (*Cannabis* spp) which have been used in traditional medicine due to their anti-inflammatory and analgesic effects. β-caryophyllene engages the cannabinoid CB2 receptors which are primarily localized in the immune and immune-derived cells (Russo, 2016). β-caryophyllene possesses analgesic activity (Fidyt et al., 2016) and, most interestingly, displays an anti-arthritic potential in an experimental rat model (Ames-Sibin et al., 2018).

Flavocoxid, a flavonoid mixture of baicalin and catechin, is a nutraceutical with a significant anti-inflammatory activity (Altavilla et al., 2009; Bitto et al., 2014), and it has been proven to be effective in OA patients (Levy et al., 2010a; Levy et al., 2010b).

Due to their “pharmacological” activities, both β-caryophyllene and flavocoxid could be good candidates for a combination drug with curcumin.

It has been previously reported that lipopolysaccharides (LPS) play an important role in the pathogenesis of rheumatoid arthritis (RA) and OA. LPS physically interact with collagen type II in the extracellular matrix (ECM) and trigger cartilage inflammation and degeneration in an *in vitro* model of human chondrocytes (Lorenz et al., 2013).

Therefore, in light of these observations, the aim of this study was to investigate the synergy potential of curcumin with both compounds in an experimental *in vitro* model of OA, based on the use of human articular chondrocytes triggered with LPS. To achieve this goal, a quantitative analysis of dose–effects relationships was used to evaluate the combined effects of the multiple compounds, using a previously validated method (Chou and Talalay, 1984; Chou, 2006).

## Materials and Methods

### Cell Cultures

Human articular chondrocytes (ScienCell, CA, USA) were cultured in the specific Chondrocyte Medium (Cat. 4651; ScienCell, CA, USA) supplemented with 1% antibiotic mixture, in 5% CO_2_ humidified incubator at 37°C. The medium was renewed every 2 days and confluent chondrocytes were trypsinized, subdivided, and re-plated. In these experiments chondrocytes from passage 3 to 7 were used.

### Cell Treatments

Chondrocytes were cultured in six well culture plates at a density of 2.5 × 10^5^ cells/well. Sixteen hours after seeding, a set of plates were treated with LPS (2 µg/ml; Escherichia coli serotype 055:B5; Sigma-Aldrich, USA) or with IL-1β (10 ng/ml) alone or in combination with curcumin (Sigma-Aldrich, USA) at doses of 0.65, 1.25, 2.5, 5, and 10 µg/ml and with flavocoxid (Primus Pharmaceuticals, AZ, USA) at doses of 4, 8, 16, 32, and 64 µg/ml. Curcumin had a purity ≥80% whereas flavocoxid had a purity ≥90% with a ratio of 4:1 in the relation between baicalin and catechin.

LPS (stock solution of 1 mg/ml) and IL-1β (stock solution 1 µg/ml) were dissolved in water whereas both curcumin and flavocoxid were dissolved in DMSO.

A further set of plates were treated with LPS (2 µg/ml) or with IL-1β (10 ng/ml) alone or in combination with curcumin at the same doses reported above and with β-caryophyllene (BCP, purity ≥90%) (Sigma-Aldrich, USA) at doses of 1.25, 2.5, 5, 10, 20 µg/ml dissolved in DMSO (10 mg/ml).BCP purity was ≥90%. Since curcumin has a half-life of about 3 h, cells underwent biochemical evaluation 4 h after the treatments.

### RNA Isolation, cDNA Synthesis, and Real-Time Quantitative PCR Amplification

Total RNA was isolated from human chondrocytes for RTqPCR analysis using Trizol Reagent Kit (Life Technologies, Monza, Italy). The first strand of cDNA was synthesized from 2.0 µg total RNA using a high-capacity cDNA Archive kit (Applied Biosystems, Carlsbad, CA). β-actin mRNA was used as an endogenous control to allow the relative quantification. RTqPCR was performed on both targets and endogenous control using SYBR Premix DimerEraser (Takara, Japan) and QuantStudio 6 Flex Real-Time PCR System (Applied Biosystems, CA, USA). The amplified PCR products were quantified by measuring the calculated cycle thresholds (CT) of targets and β-actin mRNA. The amounts of specific mRNA in samples were calculated using the 2^-ΔΔCT^ method. The mean value of investigated mRNA levels in LPS or IL-1β-stimulated chondrocytes was chosen as the calibrator, and the results are expressed as % of reduction compared to LPS or IL-1β controls. The IC_50_ value was calculated by fitting the progress curves to the four-parameter IC_50_ equation using GraphPad prism software version 5.03 (Graphpad software, CA, USA).

The oligonucleotides used as primers were as follows: IL-1β forward, 5′-GATAAGCCCACTCCTACAGCTGG-3′; IL-1β reverse, 5′-GCTTGAGAGGTGCTGATGTACC-3′, NF-κB forward, 5′-TGGAGTCTGGGAAGGATTTG-3′; NF-κB reverse, 5′-GCTTCTGACGTTTCCTCTGC -3′, STAT3 forward, 5′-TTTCACTTGGGTGGAGAAGG -3′; STAT3 reverse, 5′-GCTACCTGGGTCAGCTTCAG-3′, COL2A1 forward 5′-AGACCTGAAACTCTGCCACC-3′; COL2A1 reverse 5′-TCTCCTTGCTCTTGCTGCTC -3′, β-actin forward, 5′-TTGTTACAGGAAGTCCCTTCCC-3′; β-actin reverse, 5′-GCTTGAGAGGTGCTGATGTACC-3′.

### Determination of Drug Interaction

Chou and Talalay multiple drug effect analysis was performed and it is based on the median-effect principle (Chou and Talalay, 1984; Chou, 2006). The Median Effect Equation states that fa/fu = (D/Dm) m, where D is the dose, fa and fu are the affected and the unaffected fractions, respectively, of IL-1β mRNA expression levels, by the dose D; Dm is the dose required to produce the median effect (i.e., IC_50_), and m is the Hill-type coefficient signifying the sigmoidicity of the dose–effect curve. The dose–response curves were then plotted as log (fa/fu), with respect to log (D), to generate the Median Effect Plot.

Determination of the synergistic versus additive versus antagonistic inhibitory effects of the combined treatment of LPS-stimulated chondrocytes with curcumin and flavocoxid or with curcumin and β-caryophyllene were assessed using the Combination Index (CI), where CI < 1, CI = 1, and CI > 1 indicate synergistic, additive, and antagonistic effects, respectively. The CI was calculated as:


$$\text{CI}=\left[{\left(\text{D}\right)}_{1}/{\left({\text{D}}_{50}\right)}_{1}\right]+[\left(\text{D}\right)\;2/{\left(\text{D}50\right)}_{2}$$

where:

(D_50_)1, (D_50_)_2_ = the concentrations of curcumin and flavocoxid or β-caryophyllene that induced 50% of reduction of IL-1β mRNA expression levels (i.e., IC_50_);

(D)_1_, (D)_2_ = the concentrations of compound in combination able to induce 50% of reduction of IL-1β mRNA expression levels.

The CI index was calculated using Grafit software.

### MTT Assay

Cell viability was evaluated by MTT assay. Chondrocytes were grown and then treated with IC_50_ doses when reached confluence. In particular, flavocoxid, curcumin, flavocoxid + curcumin, β-caryophyllene, and β-caryophyllene + curcumin were tested in a 96-well plate at a density of 8 × 10^4^ cells/well for 24 h to evaluate the cytotoxic effect. The tetrazolium dye MTT 3-(4,5-dimethylthiazol-2-yl)-2,5-diphenyltetrazolium bromide (Sigma Aldrich, Milan, Italy) was dissolved in sterile filtered PBS, and 20 µl of the mixture were added into each well 5 h before the end of the 24 h of incubation. Medium was removed and the insoluble formazan crystals were dissolved with dimethyl sulfoxide (DMSO; 200 µl/well) following 5 h. The difference between the values obtained at 540 and 620 nm of absorbance was used to calculate the average of replicates and to evaluate cytotoxicity. Results were expressed as % of cell viability compared to untreated cells and reported as means and SD.

### Statistical Analysis

Results obtained from RTqPCR *were* statistically *analyzed* calculating *standard deviation* (SD). Data are expressed as the mean ± SD and the values reported are the result of at least five experiments performed in duplicate. All assays were repeated three times to ensure reproducibility.

## Results

### Curcumin and Flavocoxid Combination: Inhibitory Effect on Chondrocyte Inflammatory Phenotype and Drug Interaction Effect.

**Figure 1:**
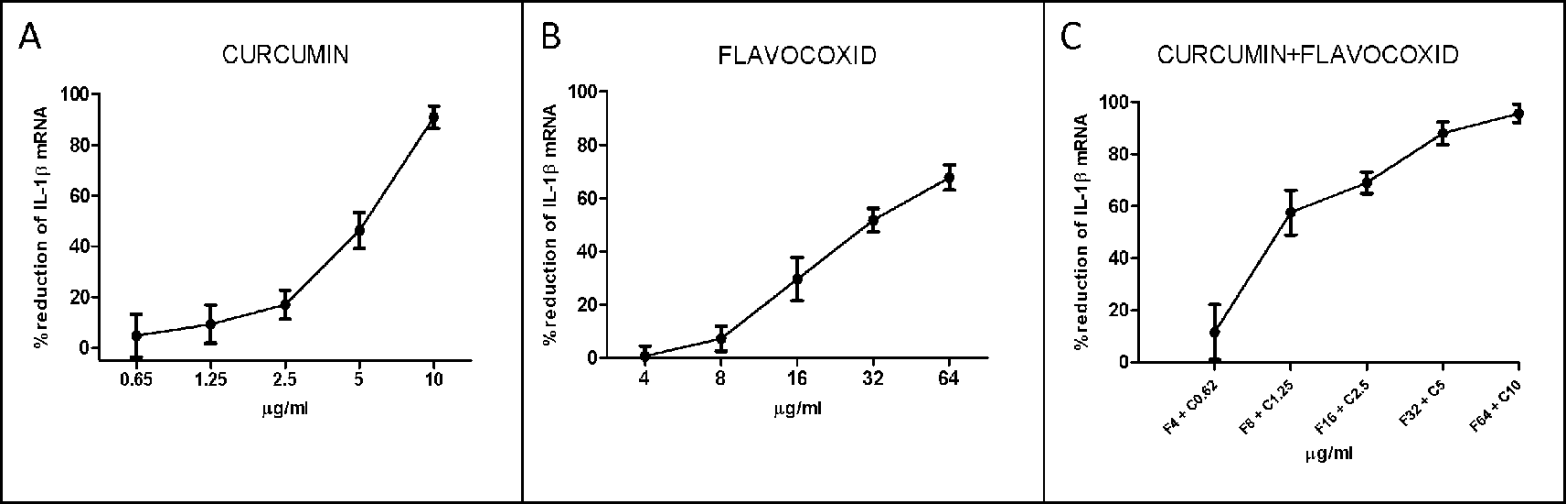
Dose–response curve for curcumin (panel **A**), flavocoxid (panel **B**), and curcumin- flavocoxid in combination (panel **C**). Data are expressed as % of reduction of IL-1β mRNA levels.

**Figure 2:**
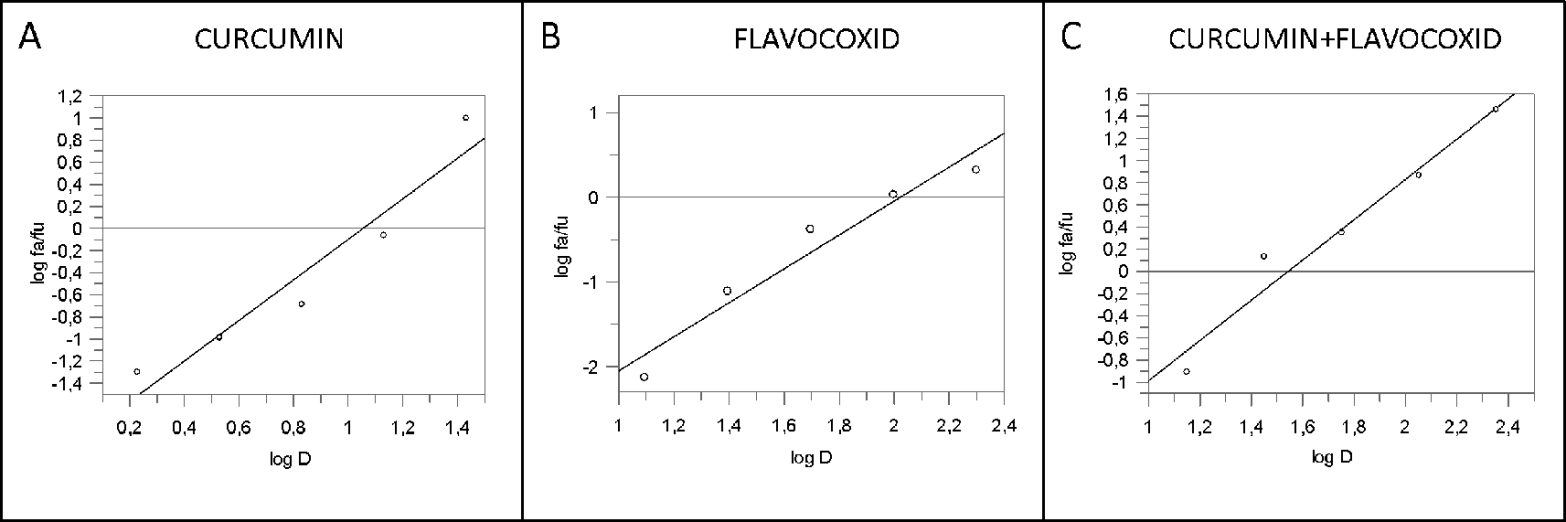
Median effect plot for curcumin (panel **A**), flavocoxid (panel **B**), and curcumin -flavocoxid in combination (molar ratio, 1:7.3) (panel **C**). D is the dose, f_a_ and f_u_ the affected and the unaffected fraction, respectively by the dose D.

IL-1β mRNA expression was used as a read-out of the inflammatory phenotype triggered by LPS in human chondrocytes. Both curcumin and flavocoxid reduced IL-1β mRNA in a dose-dependent manner, as shown by the curve of five different doses (**Figure 1**). IC_50_ values derived from the dose–response curves were 91.2 µM for favocoxid and 14.28 µM for curcumin, respectively (**Figure 1**). Combination of curcumin and flavocoxid also inhibited the chondrocyte inflammatory phenotype in a dose dependent manner and the IC_50_ value was 26.3 µM (**Figure 1**). According to Chou and Talalay method (Chou and Talalay, 1984; Chou, 2006), the Median Effect Equation indicates that f_a_/f_u_ = (D/D_m_)^m^, where D is the dose, f_a_ and f_u_ are the fractions of protected and not protected cells by the dose D; D_m_ is the dose required to produce the median effect (i.e. IC_50_) and m represents the Hill-type coefficient signifying the dose–response curve (Chou and Talalay, 1984; Chou, 2006). Thereafter, the dose–response curve was plotted (**Figure 1**) as log (f_a_/f_u_) respect to log (D) to generate the median effect Plot (**Figure 2**). Summarizing the results, the comparison of the IC_50_ of curcumin, flavocoxid and the combination curcumin+flavocoxid and their related m for each median effect plot showed IC_50_ = 14.28 and m_1_ = 1.835 for curcumin; IC_50_ = 91.2 µM and m_1_ = 2.005 for flavocoxid; and IC_50(1,2)_ = 26.3 µM and m_1,2_ = 1.816 for curcumin-flavocoxid combination (molar ratio, 7.3:1).

**Figure 3:**
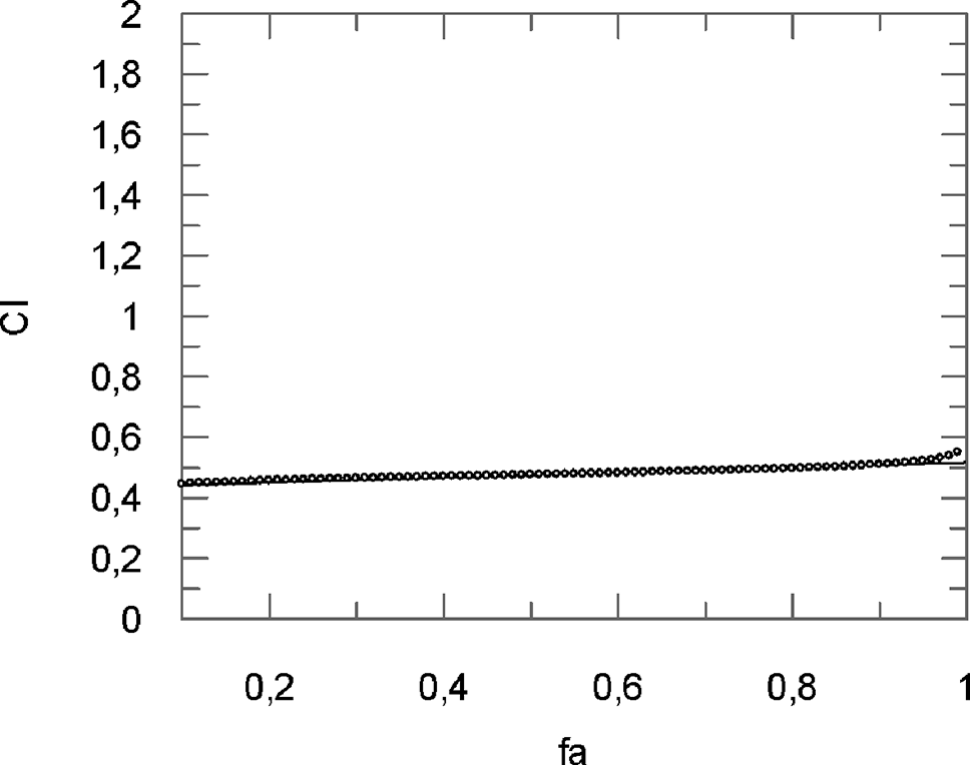
Computer-generated graphical presentation of the combination index (*CI*) *vs* the fraction affected, i.e., the effect of reduction of IL-1β mRNA levels exerted by a mixture of curcumin-flavocoxid (molar ratio, 1:7.3) in chondrocytes.

The type of interaction between curcumin and flavocoxid was investigated by the Chou and Talalay analysis (Chou and Talalay, 1984; Chou, 2006). Moreover, the combination index (CI) was used to clarify the nature of the interactions (synergistic *versus* additive *versus* antagonistic inhibitory effects, respectively. The CI was calculated as follows:


$$\text{CI}=\left[{\left(\text{D}\right)}_{1}/{\left({\text{D}}_{50}\right)}_{1}\right]+[\left(\text{D}\right){\;}_{2}/{\left({\text{D}}_{50}\right)}_{2}$$

where (D_50_)_1_ and (D_50_)_2_ represented the concentration of curcumin and flavocoxid able to cause a 50% of cell protection. Furthermore, (D)_1_ and (D)_2_ indicated the concentration of curcumin and flavocoxid in combination to induce 50% of cell protection. The CI was then measured using a Grafit software (**Figure 3**).

The obtained results clearly indicate that CI was < 1 at all the affected fraction, thus suggesting the presence of a synergistic effect (**Table 1**).

**Table 1:** Calculated values for the combination index of flavocoxid-curcumin in combination (molar ratio, 1:7.3) for the reduction of IL-1β mRNA levels in chondrocytes.

Fraction affected (f_a_)	Combination index (CI)	Diagnosis of combined effect
0.05	0.43	Synergism
0.10	0.44	Synergism
0.20	0.45	Synergism
0.30	0.46	Synergism
0.40	0.47	Synergism
0.50	0.47	Synergism
0.60	0.48	Synergism
0.70	0.49	Synergism
0.80	0.50	Synergism
0.90	0.51	Synergism
0.95	0.52	Synergism

The combination index was generated on the bases of the parameters obtained from the median effect plot.

### Curcumin and β-Caryophyllene Combination: Inhibitory Effect on Chondrocyte Inflammatory Phenotype and Drug Interaction Effect

**Figure 4:**
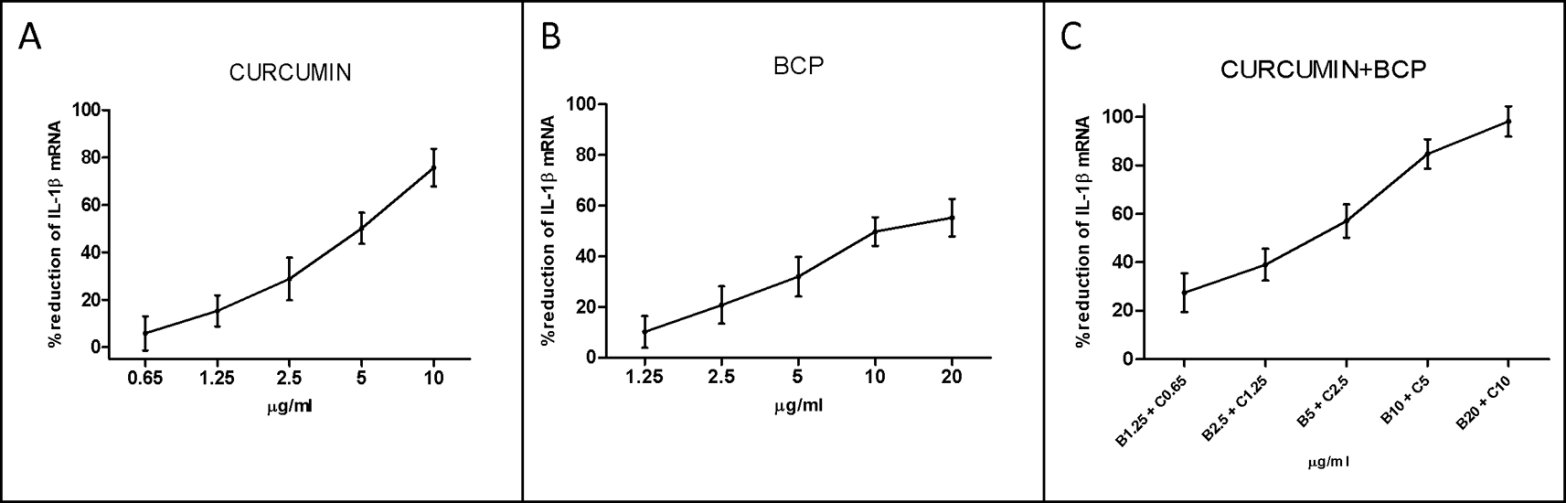
Dose-response curve for curcumin (panel **A**), BCP (panel **B**) and BCP-curcumin in combination (panel **C**). Data are expressed as % of reduction of IL-1β mRNA levels.

**Figure 5:**
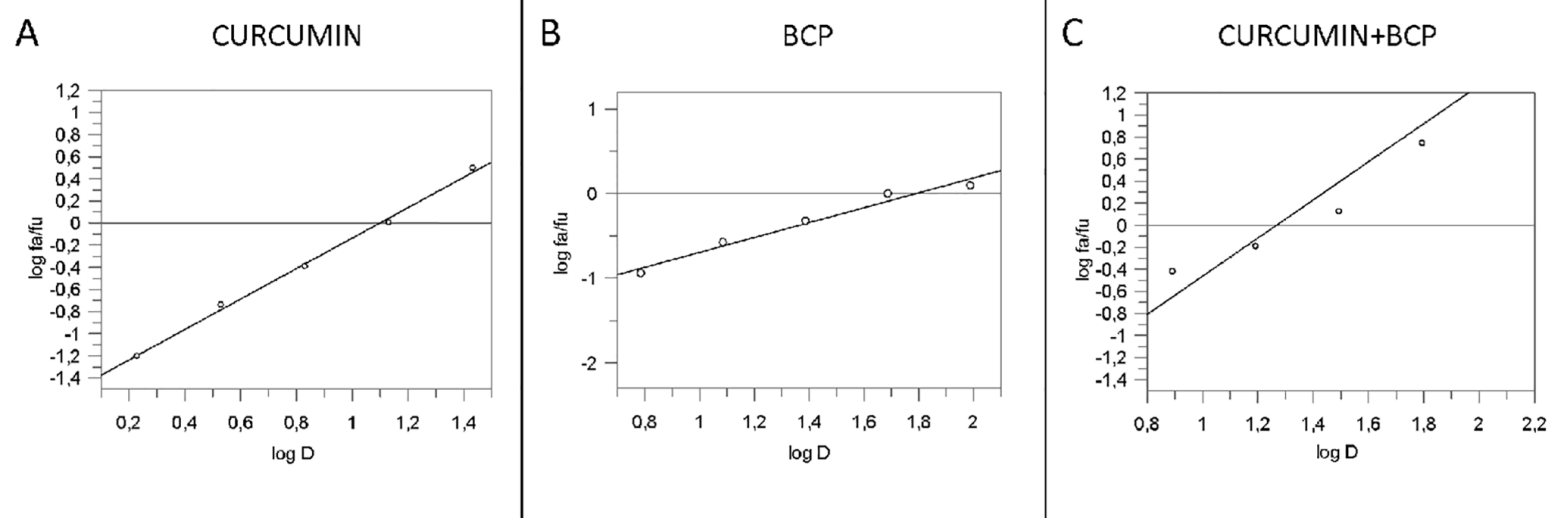
Median effect plot for curcumin (panel **A**), BCP (panel **B**) and curcumin-BCP in combination (molar ratio, 1:3.6) (panel **C**). D is the dose, f_a_ and f_u_ the affected and the unaffected fraction, respectively by the dose D.

An overlapping approach was used to investigate the nature of drug interaction between curcumin and β-caryophyllene. **Figure 4** shows dose–response curve plotted as log (f_a_/f_u_) compared to log (D) to generate the median effect Plot (**Figure 5**). Comparison of the IC_50_ of curcumin, β-caryophyllene and the combination of curcumin +β-caryophyllene and their related m for each median effect plot showed IC_50_ = 13.57 and m_1_ = 1.38 for curcumin; IC_50_ = 48.93 µM and m_1_ = 0.88 for β-caryophyllene; and IC_50(1,2)_ = 28.28 µM and m_1,2_ = 1. 73 for curcumin-BCP combination (molar ratio, 3.6:1).

**Figure 6:**
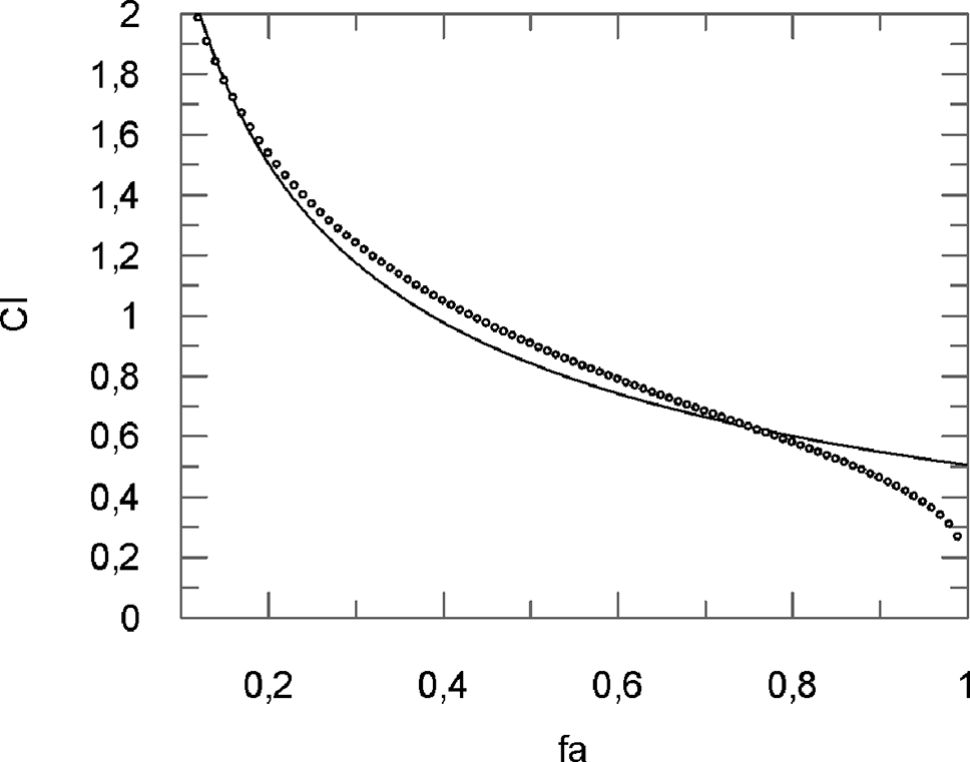
Computer-generated graphical presentation of the combination index (CI) *vs* the fraction affected, i.e. the effect of reduction of IL-1β mRNA levels exerted by a mixture of curcumin-BCP (molar ratio, 1:3.6) in chondrocytes.

The analysis of the CI (**Figure 6** and **Table 2**) shows for f_a_ ranging from 0.10 to 0.30 an antagonistic effect, for f_a_ = 0.40 an additive effect, while for the most relevant f_a_ values, i.e., fa ranging from 0.50 to 0.90, a synergistic effect obtained.

**Table 2:** Calculated values for the combination index of curcumin-BCP in combination (molar ratio, 1:3.6).for the effect of reduction of IL-1β mRNA levels in chondrocytes.

Fraction affected (f_a_)	Combination index (CI)	Diagnosis of combined effect
0.10	2.1677	antagonistic
0.20	1.5360	additive
0.30	1.2389	additive
0.40	1.0480	additive
0.50	0.9053	synergistic
0.60	0.7876	synergistic
0.70	0.6820	synergistic
0.80	0.5783	synergistic
0.90	0.4609	synergistic

The combination index was generated on the bases of the parameters obtained from the median effect plot.

### Flavocoxid, Curcumin and β-Caryophyllene Alone or in Combination Do Not Affect Cell Viability

**Figure 7:**
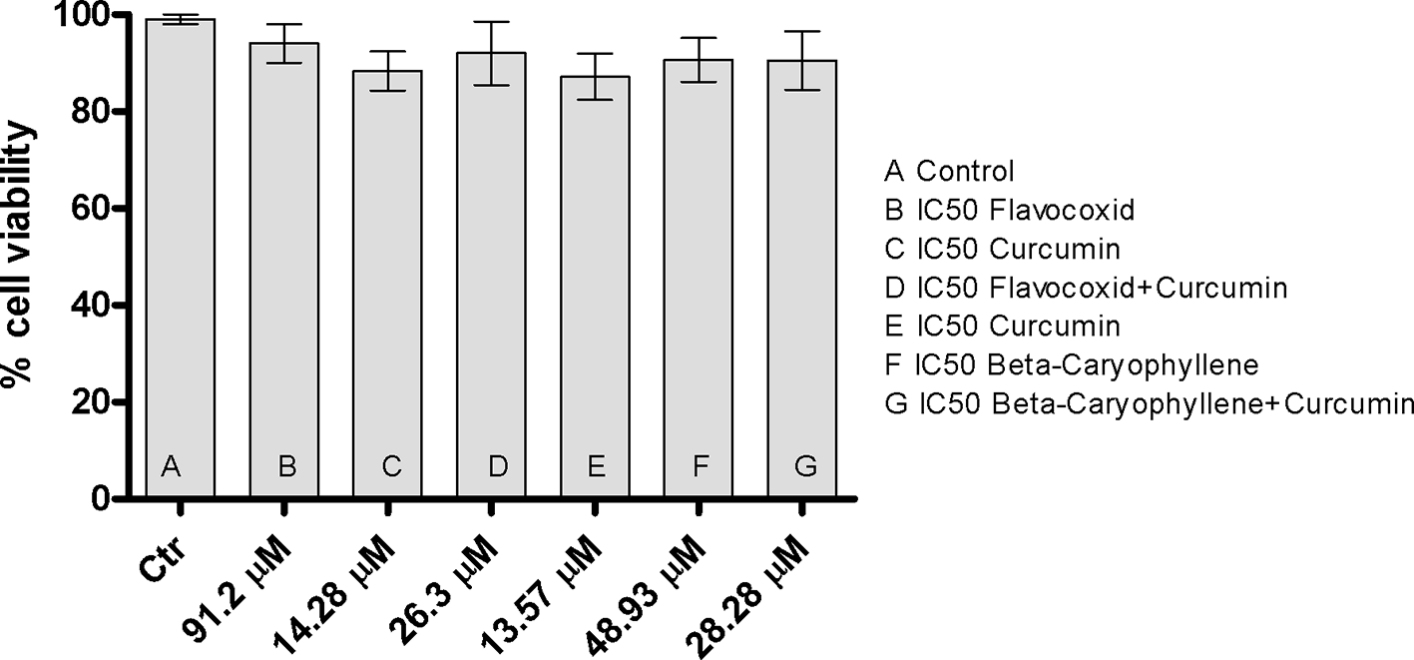
The graph shows cytotoxicity assay at 24 h in control cells (chondrocytes) and in chondrocytes treated with IC_50_ doses of flavocoxid, BCP, curcumin and flavocoxid+curcumin and BCP+curcumin. All IC_50_ doses were tested in duplicate.

One hundred percent of viability was observed on control cells following 24 h. The incubation with the IC_50_ concentrations of flavocoxid, curcumin, flavocoxid + curcumin, β-caryophyllene, and β-caryophyllene + curcumin did not affect chondrocytes viability, thus demonstrating that these natural products alone or in combination do not have a cytotoxic effect and that the synergistic effect does not affect cell viability (**Figure 7**).

### Flavocoxid, β-Caryophyllene, and Curcumin Inhibit the Transcriptional Factors NF-κB and STAT3

**Figure 8:**
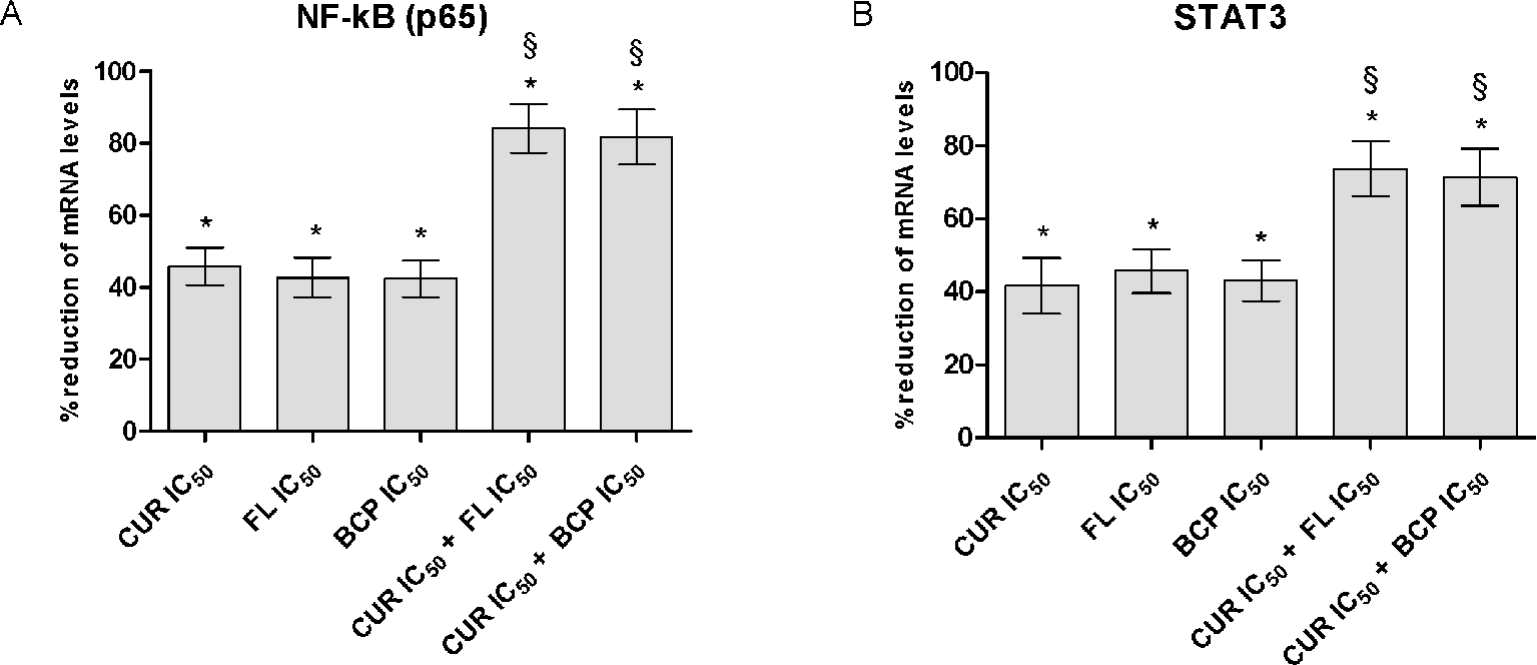
NF-kB (panel **A**) and STAT3 (panel **B**) mRNA expression evaluated by RTqPCR. Data are expressed as % of reduction of mRNA levels vs LPS. Values are expressed as the mean ± SD. *p < 0.001 versus LPS (not shown); §*p* < 0.001 versus LPS + CUR (curmin) IC_50_, LPS + FL (flavocoxid) IC_50_ and LPS + BCP (β-caryophyllene) IC_50_.

The IC_50_ doses were used to confirm flavocoxid, β-caryophyllene, and curcumin mechanism of action on inflammatory phenotype. Flavocoxid, β-caryophyllene, and curcumin reduced both NF-κB and STAT3 mRNA expression. In particular, interestingly, the combination of IC_50_ concentration of flavocoxid and curcumin and β-caryophyllene and curcumin caused a greater reduction of NF-κB and STAT3 mRNA expression compared to flavocoxid, β-caryophyllene, and curcumin alone, thus confirming the synergistic anti-inflammatory effect of the combinations (**Figure 8**).

### Flavocoxid, β-Caryophyllene, and Curcumin Restore Type II Collagen Expression in Human Chondrocytes Stimulated With LPS and IL-1β

**Figure 9:**
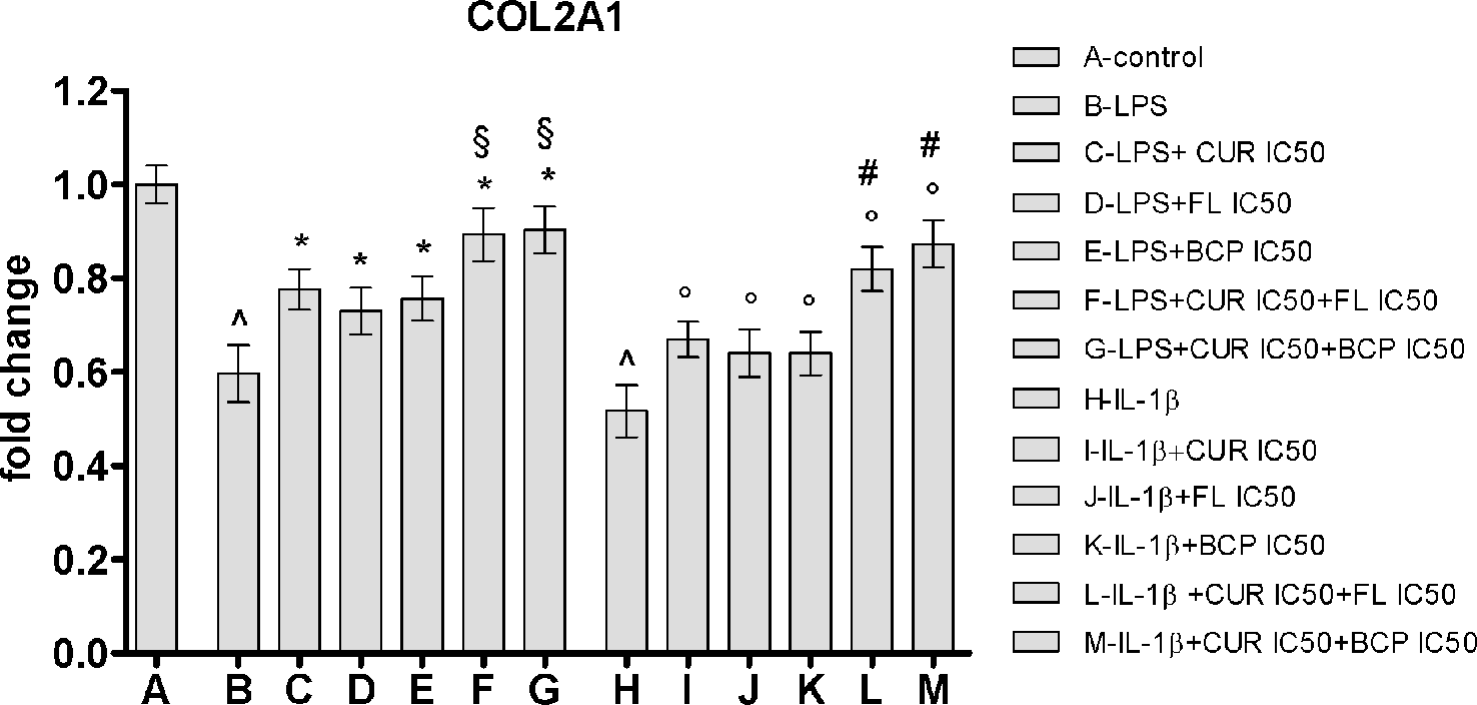
COL2A1 mRNA expression evaluated by RTqPCR. Data are expressed as fold change versus control. Values are expressed as the mean ± SD. ^*p* < 0.001 versus control; *p < 0.001 versus LPS; §*p* < 0.001 versus LPS + CUR (curmin) IC_50_, LPS + FL (flavocoxid) IC_50_ and LPS + BCP (β-caryophyllene) IC_50_. °*p* < 0.001 versus IL-1β; #*p* < 0.001 versus IL-1β + CUR (curmin) IC_50_, IL-1β + FL (flavocoxid) IC_50_ and IL-1β + BCP (β-caryophyllene) IC_50_.

IC_50_ doses effect was evaluated on a component of cartilage matrix, highly expressed in chondrocytes, the type II collagen. In this experimental setting, the inflammatory response was induced by two different stimulation, adding LPS or IL-1β. Both pro-inflammatory agents caused the reduction of the type II collagen expression in human chondrocytes compared to control cells. Curcumin, flavocoxid, and β-caryophyllene were partially able to restore the mRNA control levels. Notably, the effect of IC_50_ concentration of the combinations on COL2A1 mRNA expression was significantly higher compared to flavocoxid, β-caryophyllene and curcumin alone, thus suggesting that the synergistic activity of the combinations may also be effective on articular cartilage (**Figure 9**).

## Discussion

In clinical practice the goals of a combination drug treatment are to obtain a synergistic therapeutic effect, to reduce the dose, to minimize toxicity, to achieve a rapid onset of action, and to insure a long-lasting therapeutic effect without the occurrence of tolerance against the curative activity.

These features are particularly requested in the treatment of diseases such as cancer (Piccolo et al., 2015; Eitsuka et al., 2016; Losson et al., 2016; Zhang et al., 2018) and AIDS (Schader et al., 2011; Excler et al., 2011; Brown et al., 2015; He et al., 2018). However, chronic inflammatory conditions, such as RA and OA may require the same approach: in fact due the clinical characteristics of the disease and to the potential toxicity of available pharmacological treatments, patients would benefit from therapeutic strategies that maximize the clinical efficacy and, at the same time, minimize the associated drug adverse events (Kwon et al., 2018; Lu et al., 2018; Scheinberg et al., 2018; Smith and Cooper-DeHoff, 2018).

Curcumin has been shown to exert a potent anti-inflammatory activity (Aggarwal et al., 2013), with an interesting translational potential.

Meta-analyses of eight randomized clinical trials enrolling more than 800 patients suffering from OA clearly pointed out and confirmed the significant efficacy of curcumin (at the dose of about 1000 mg/day) in the management of the inflammatory disease (Daily et al., 2016; Onakpoya et al., 2017). However, because of its erratic bioavailability that weakens the clinical efficacy, several strategies have been used to increase curcumin poor pharmacokinetics profile and the most successful approach was to use nanoparticle-based formulation and liposome-encapsulated curcumin with the aim of maximize the clinical efficacy. Besides this, it has been proposed that a fixed combination of curcumin with other active phytochemicals might overcome the *in vivo* reduced efficacy of curcumin by creating a multi-step therapy with a positive interaction on the several targets of the underlining disease (OA). This has been recently confirmed by a randomized double-blind placebo controlled trial that exploited the safety and the efficacy of curcumin in combination with the anti-inflammatory phytochemical boswellic acid in patients suffering from OA (Haroyan et al., 2018). Indeed, the idea of a multi-target therapy with a combination of natural products have been proposed (Efferth and Koch, 2011) and synergy between them has been suggested as the “rationale” for this therapeutic strategy with a particular emphasis on the potential ability of curcuminoids to synergize with other phytochemicals (Comblain et al., 2015;Panossian et al., 2015).

Synergy concept, as well as the methods used to demonstrate synergy, has been questioned (Chou and Talalay, 1984). In this context, the *p* value has been often used to claim synergy. However, a combined effect greater than each drug alone does not necessarily mean synergism, as claimed in a previous synergy study with curcumin (Comblain et al., 2015). This increased effect can be due to an additive effect or in addition may represent a potentiation of the less effective “drug.” The additive effect of two drugs does not represent the result of the arithmetic sum of the two drugs. If the first drug and the second drug exert an inhibitory effect of 25% and 35%, respectively, the additive effect is not 60%, because if they inhibit 70% and 80%, respectively, the additive effect cannot be 150%. Therefore, it is clear that in the absence of a clear cut “evidence of synergy” validated by a theory scientifically accepted and widely used, it is hard to make synergy claims either in scientific papers or in patent application.

The Chou-Talalay method has been used worldwide for drug combination investigation and their synergy evaluation (Chou and Talalay, 1984; Chou, 2006). It is mainly based on the median-effect equation and results in the combination index (CI) theorem where CI = 1 denotes an additive effect, CI < 1 synergism and C > 1 antagonism when two compounds are considered for a possible combination. As far as we know, the potential synergy of curcumin with other natural products has not been previously exploited using this method, widely used for drug combination studies. A synergistic action for the reduction of the inflammatory phenotype in human chondrocytes was observed from 10% to 90% for the combination curcumin-flavocoxid, and from 50% to 90% for the combination curcumin-β-caryophyllene.

IC_50_ doses of either flavocoxid, BCP and curcumin alone or in combination were safe and did not affect cell viability. This points out that the synergy between the several compounds does not cause an enhancement of the potential toxicity.

Moreover, IC_50_ doses reduced the transcription factors NF-κB and STAT3 mRNA expression and interestingly the effects of the combinations were greater than the natural products alone, thus suggesting that the site where the synergy takes place could be at the signal transduction level.

Importantly, the synergistic effect of the combinations has been demonstrated exploring an important articular cartilage marker: type II collagen. One of the most important feature is the impairment of the articular cartilage matrix in OA and previous studies showed that curcumin not only has anti-inflammatory effects, but also stimulates type-II collagen expression (Shakibaei et al., 2005; Henrotin et al., 2010). Type II collagen mRNA expression was reduced in LPS and IL-1β stimulated chondrocytes, thus demonstrating that the stimulation with LPS or IL-1β similarly impaired human chondrocytes. More significantly, IC_50_ concentration of the combinations increased type II collagen mRNA expression, thus confirming the synergistic positive effect of Flavocoxid, BCP and curcumin.

This synergism strongly suggests the potential of a dual combination of these compounds for the management of OA and unmasks a new feature of these natural products.

## Author Contributions

AD’A and NI conceived and designed the study. GPa, FM, VS, and GPi performed the experimental procedures. RE, AB, MA, GC, LM, and VA analyzed and interpreted the data. FS and DA led the design and drafted the paper.

## Conflict of Interest

Author DA is co-inventor on a patent related to curcumin synergy in anti-inflammatory formulations. Author FS has received research support from Primus Pharmaceuticals for work on genistein.

The remaining authors declare that the research was conducted in the absence of any commercial or financial relationships that could be construed as a potential conflict of interest.
