# Corrigendum: Exploiting curcumin synergy with natural products using quantitative analysis of dose-effect relationships in an experimental *in vitro* model of osteoarthritis

**DOI:** 10.3389/fphar.2022.1073679

**Published:** 2022-11-07

**Authors:** Angela D’Ascola, Natasha Irrera, Roberta Ettari, Alessandra Bitto, Giovanni Pallio, Federica Mannino, Marco Atteritano, Giuseppe M. Campo, Letteria Minutoli, Vincenzo Arcoraci, Violetta Squadrito, Giacomo Picciolo, Francesco Squadrito, Domenica Altavilla

**Affiliations:** ^1^ Department of Clinical and Experimental Medicine, University of Messina, Messina, Italy; ^2^ Department of Chemical, Biological, Pharmaceutical and Environmental Sciences, University of Messina, Messina, Italy; ^3^ Department of Biomedical and Dental Sciences and Morphofunctional Imaging, University of Messina, Messina, Italy

**Keywords:** synergy, curcumin, flavocoxid, beta-caryophyllene, inflammation

In the published article, the **Conflict of Interest statement** was incorrectly filed. The correct Conflict of Interest statement appears below.

The authors apologize for this error and state that this does not change the scientific conclusions of the article in any way. The original article has been updated.

